# Kinematic changes in goal-directed movements in a fear-conditioning paradigm

**DOI:** 10.1038/s41598-021-90518-7

**Published:** 2021-05-27

**Authors:** Yuki Nishi, Michihiro Osumi, Masahiko Sumitani, Arito Yozu, Shu Morioka

**Affiliations:** 1 Department of Neurorehabilitation, Graduate School of Health Sciences, Kio University, 4-2-2 Umaminaka, Koryo-cho, Kitakatsuragi-gun, Nara, 635-0832 Japan; 2 Department of Rehabilitation Medicine, Nishiyamato Rehabilitation Hospital, Nara, Japan; 3 Neurorehabilitation Research Center, Kio University, Nara, Japan; 4 Department of Pain and Palliative Medicine, The University of Tokyo Hospital, Tokyo, Japan; 5 Department of Precision Engineering, The University of Tokyo, Tokyo, Japan

**Keywords:** Human behaviour, Pain

## Abstract

In individuals with a musculoskeletal disorder, goal-directed reaching movements of the hand are distorted. Here, we investigated a pain-related fear-conditioning effect on motor control. Twenty healthy participants (11 women and 9 men, 21.7 ± 2.7 years) performed a hand-reaching movement task. In the acquisition phase, a painful electrocutaneous stimulus was applied on the reaching hand simultaneous with the completion of reaching. In the subsequent extinction phase, the task context was the same but the painful stimulus was omitted. We divided the kinematic data of the hand-reaching movements into acceleration and deceleration periods based on the movement-velocity characteristics, and the duration of each period indicated the degree of impairment in the feedforward and feedback motor controls. We assessed the wavelet coherence between electromyograms of the triceps and biceps brachii muscles. In the acquisition phase, the durations of painful movements were significantly longer in both the acceleration and deceleration periods. In the extinction phase, painful movements were longer only in the acceleration period and higher pain expectation and fear were maintained. Similarly, the wavelet coherence of muscles in both periods were decreased in both the acquisition and extinction phases. These results indicate that negative emotional modulations might explain the altered motor functions observed in pain patients.

## Introduction

Among patients with a musculoskeletal disorder, repeated painful experiences during and immediately after goal-directed reaching movements of the hand frequently result in fear of movement. Fear of movement has been defined as an excessive, irrational and debilitating fear of carrying out a physical movement due to a feeling of vulnerability to a painful injury or reinjury^1^, and it is associated with critical clinical problems such as disability, impaired health-related quality of life, and severe pain^2–4^.

When fear of movement emerges, the central nervous system adapts the body's movement strategies to avoid pain and minimize its harmful consequences^5, 6^. However, protective movement strategies such as freezing, bradykinesia, and muscle co-contractions can result in or prolong physical impairments and lead to greater disability^7, 8^. These protective motor controls have been reported to be associated with not only pain but also pain expectancy^9^.

Among the protective motor controls, the co-contraction between agonist/antagonist muscles in particular can change the coherence of the muscle contractions in a specific frequency band. Analyses of the specific band coherence can provide information regarding the origin of a common oscillatory drive to different motor units, reflecting the activity of the corticospinal tract^10, 11^. In particular, the β-band coherence of the muscular co-contractions reflects the cortical processing of the primary motor cortex, and thus reduced β-band coherence indicates less-efficient motor unit recruitments^12–14^. Analyses of the factors that change in agonist/antagonist muscles will contribute to our understanding of the processes of changes in motor control related to pain and/or fear, but these factors currently remain unclear. In this context, analysis of the coherence of muscle contractions may provide valuable insights into abnormal motor control in pain patients.

Behavioral effects of pain-related fear have been investigated using a fear-conditioning paradigm^15^. In this paradigm, the experimental stimulus that elicits pain perception and fear responses is called the unconditioned pain stimulus (Pain-US). The neutral stimulus paired with the Pain-US is called the conditioned stimulus (CS+). After a CS+ is paired with a Pain-US, the CS+ becomes able to elicit a fear response by itself. In contrast, a control stimulus (CS−) that is never paired with the US fails to elicit such a conditioned fear response. In addition, the process in which the fear response to the CS+ lessens when the Pain-US is removed is called “extinction”.

We conducted the present study to determine whether the fear and pain expectancy can change an individual's motor control and the co-contraction coherence in goal-directed reaching. In regard to motor control, goal-directed reaching movements of the hand can be divided into feedforward and feedback motor controls based on the characteristics of the movement velocity^16–18^. We also sought to clarify the changes in these two types of motor control in a fear-conditioning paradigm. We conducted a wavelet coherence analysis of our pain-related fear-conditioning paradigm to clarify changes in the output of agonist/antagonist muscles related to corticospinal excitability. We hypothesized that motor behaviors of feedforward and feedback motor controls are changed following fear acquisition and diminish when the pain-US is omitted.

## Participants and methods

### Participants

Twenty right-handed healthy participants were recruited at Kio University: 11 women and 9 men; mean age: 21.7 ± 2.7 (SD) years. The exclusion criteria were the presence of neurological diseases; any current or past psychiatric disorders including clinical depression; hearing problems; a painful upper limb or related problems including acute or chronic pain; a cardiac pacemaker or any other electronic medical devices; and the presence of any other severe medical condition. The study was approved by the Ethics Committee of Kio University, Health Science Graduate School (H28-43), and the study protocol conformed to the Declaration of Helsinki. Before participating, each participant signed an informed consent form emphasizing that their participation was completely voluntary and that they could decide not to participate at any time during the experiment.

### Stimulus material

The unconditioned stimulus was a painful electrocutaneous stimulus (Pain-US) (100-ms duration, 50 Hz). This electrical stimulus was administered to the participant's dominant palm by a commercial constant current stimulator (SEN-8203; Nihon Kohden, Tokyo) through two surface electrodes (1 cm diameter, 2 cm inter-electrode distance). The location of the stimulation site remained the same throughout the experiment.

During the calibration procedure, each participant received electrocutaneous stimuli, starting with an intensity of 0.2 mA and increasing in steps of 0.4 mA, and was asked to rate each stimulus on an 11-point numerical rating scale (NRS) ranging from 0 (“I feel something but it is not painful, merely a sensation”) up to 10 (“This is the worst tolerable pain I can imagine”). Once this rating was provided, the experimenter presented the next stimulus intensity. This calibration procedure was adapted from the procedure in a study by Gatzounis et al.^19^. The series was ended when the participant had rated the last stimulus as an 8 (“significantly painful and demanding some effort to tolerate”) on the NRS. This procedure to determine the stimulus intensity level for individual participants was repeated five times, and the average of the five electrical stimulus intensities that were rated an 8 was used as the painful stimulation in the acquisition phase for the respective participant. The mean intensity of the pain-US was 6.51 ± 0.85 (SD) mA for the entire series of participants.

### Apparatus and procedure

The participants performed a hand-reaching movement toward a 3-cm-wide, 3-cm-high and 4-cm-long object. The start position was set at 20 cm away from the participant, and the object was located 30 cm further away from the start position; i.e., the distance from the participant to the object was 50 cm. The start position and the object were aligned with the participant's sagittal body-midline axis. The participant was required to first put his or her dominant hand on the start position (which was oriented in an anteroposterior direction of the sagittal body-midline axis) and then move the hand from the start position to reach and grasp the object (goal position).

We used a VICON Motion System (Oxford Metrics, Oxford, UK) for recording kinematic data. This system consists of six infrared capture cameras (sampling rate: 120 Hz) and a data station in which the information is gathered. A passive 14-mm reflective marker was placed on the participant's dominant wrist. The electromyographic (EMG) (Trigno; Delsys, Boston, MA) activity was recorded from the biceps brachii and triceps brachii of the participant's dominant arm. The EMG signal was digitized at 1926 Hz. Prior to placing the electrodes, the skin was prepared by shaving the area and cleaning it with alcohol in order to reduce impedance.

Each experiment took 90 min and had three phases: a practice, an acquisition, and an extinction phase. The number of trials in each phase was based on the study by Meulders et al.^20^.

### Movement paradigm

**Figure 1:**
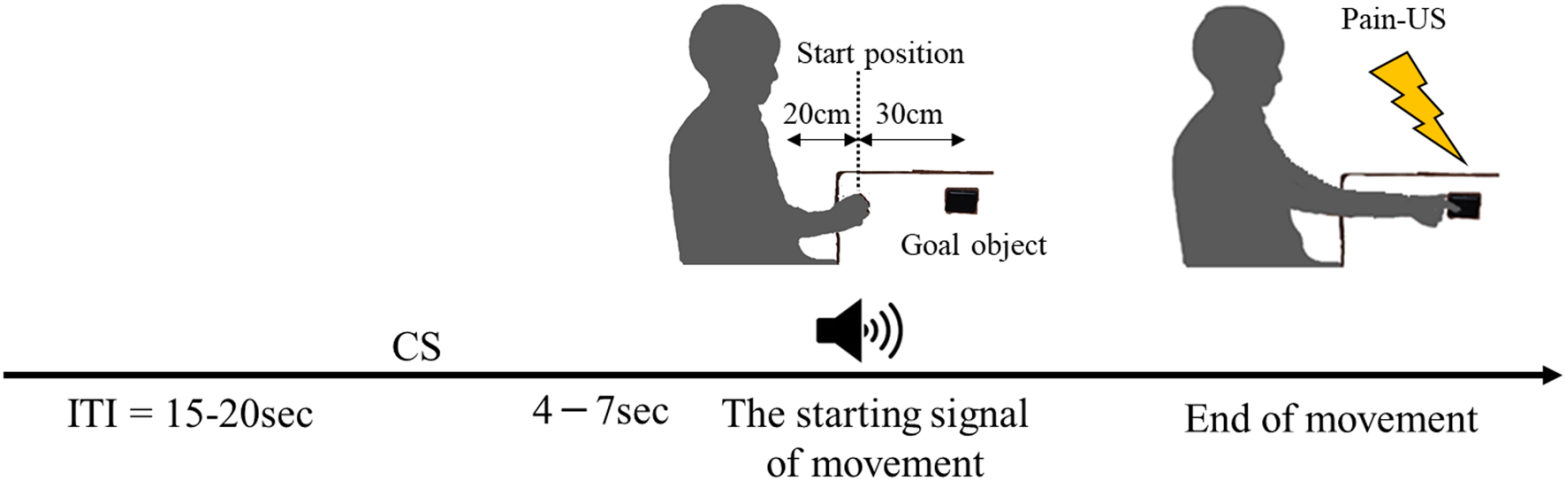
Flow chart of the experimental task. CS: conditioned stimulus. *Lightning bolt:* The presentation of the painful electrocutaneous stimulus (pain-US).

In the experimental trial sessions, we asked the participants to execute the goal-directed hand-reaching movement. For this study, we defined the fear-conditioning settings as follows: the unconditioned stimulus (US); a painful electrical stimulus (Pain-US), and the conditioned stimulus (CS); Two types of beep sounds. One beep was used for the conditioned stimulus [CS+] with the Pain-US, and the other beep represented the conditioned stimulus with no Pain-US [CS−]. The CS+ and CS− beeps were set at 300 Hz and 1000 Hz, respectively and counterbalanced across participants. The CS+ and CS− trials were randomly assigned with the restriction of no more than two consecutive trials under the same conditions. The CSs were delivered immediately after an inter-trial interval of 15–20 s via speakers (Z120BW; Logicool, Tokyo). The duration of each CS was 100 ms. In this paradigm, the starting signal chimed in at 4–7 s after the CS+ or CS−, and then the participant started the hand-reaching movement. The Pain-US was delivered immediately after the participant's completion of each goal-directed reaching movement (Fig. 1). The timing of the Pain-US delivery was the same as that described in previous reports using the fear-conditioning paradigm^15, 21^.

### Practice phase

**Figure 2:**
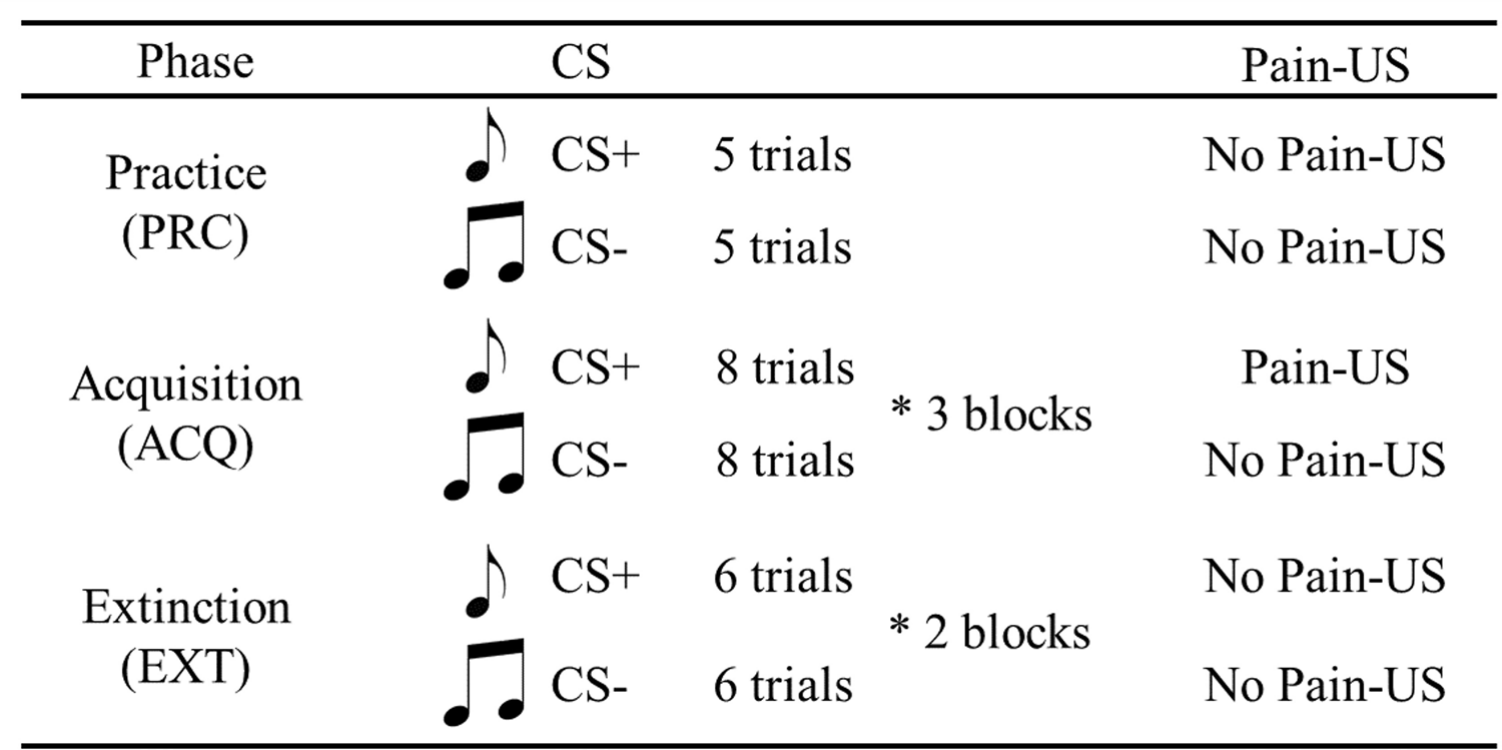
Study design. CS+ : The movement that is paired with the pain-US only during the acquisition phase. CS− : The movement that is never paired with the pain-US.

This phase was comprised of 10 trials: 5 trials delivering the CS+ and 5 trials delivering the CS−. No Pain-US was delivered during this phase (Fig. 2). The participants were verbally instructed to perform the reaching movement after hearing the starting signal (which indicated the beginning of a trial). In the practice phase, we intended to measure the individual's reaching movements in the absence of pain expectancy/fear of movements. We did not explain to the participants that the pain-US was paired with a CS prior to the practice phase, because with such an explanation the participants might have expected a pain stimulus and felt fear, and this psychological factor could have affected their movements in the practice phase.

### Acquisition phase

Each participant performed a total of 48 trials in the acquisition phase. The 48 trials consisted of 3 blocks of 16 trials in which 8 CS+ and 8 CS− were delivered (Fig. 2). The painful electrocutaneous stimulus (Pain-US) was applied to the participant's reaching hand at the completion of the participant's reaching movement. In the acquisition phase, the CS+ was paired with the Pain-US, and the CS− was never paired with the Pain-US. Thus, the CS+ and CS− were regarded as unsafe and safe contextual cues, respectively. After each trial, the participants rated their pain-related fear and their expectation of pain (the details of these ratings are described below).

### Extinction phase

During the extinction phase, the No Pain-US was administered. The participants performed 2 blocks of 12 trials in which 6 CS+ and 6 CS− were delivered (Fig. 2). After each trial, the participants rated their pain-related fear and expectation of pain.

### Outcome measures

#### Self-report measures

After each trial (CS+ /CS−) of acquisition and extinction phases, the participants answered two questions which retrospectively assessed how they felt during the performance of the movements: (1) “To what extent were you afraid that the movement was going to be painful?” (fear of pain-related movement) and (2) “To what extent did you expect an electrical stimulus when moving?” (pain expectancy). Both questions were scored on an 11-point Likert scale ranging from “not at all” (0) to “very much” (10).

#### Kinematic data

We used Matlab 2018b software (MathWorks, Natick, MA) to analyze the recorded kinematic data. The movement onset was determined for each trial by an algorithm that determined when the forward velocity rose 10-SD above the pre-movement mean velocity, and then a backwards local minima search identified the onset^22^. We divided the reaching trajectory into the acceleration period and the deceleration period. Based on our previous study^23^, we defined the acceleration period as the time point from the movement onset until the time point at which the velocity of the participant's limb reached a maximum value. The deceleration period was defined as starting immediately after the acceleration period and lasting until the velocity fell below the same threshold of the movement onset (i.e., 10-SD above the pre-movement mean velocity). We calculated the duration of the acceleration period and the duration of the deceleration period (unit: seconds) in each trial.

#### EMG analysis

We also determined the muscle co-contraction between the biceps and triceps brachii muscles during the reaching task. The onset of EMG activity was determined as > 10 SDs of the root mean square of the EMG activity of the triceps during the pre-movement.

To investigate the details of the co-contractions, we performed a wavelet coherence analysis. Wavelet coherence is considered efficient and reliable for detecting the synchronizing activity between two time series^8, 13, 24^. In this study, we used wavelet coherence to test the linear dependency of two sequences of surface EMGs in the time–frequency domain. We applied the Morlet wavelet function for the transformation. The EMG–EMG coherence was estimated in the following four frequency bands: δ and θ (0–5 Hz), α (6–15 Hz), β (16–35 Hz), and γ (36–60 Hz). We focused our analyses on the α-, β-, and γ-bands. The coherence values at the δ and θ-bands were not examined in detail because the coherence in this frequency band is not thought to originate from the corticospinal system^25^. The magnitude-squared coherence was computed from the unrectified high-pass filtered (3 Hz, 4th-order zero-lag Butterworth) EMG time series. The EMG–EMG coherence value was calculated as the volume under the magnitude-squared coherence values in the time window of interest in which a significant correlation between the EMG time series was detected on the wavelet cross-spectrum^26^. Each frequency band was calculated by subtracting the frequency band of the wavelet coherence in the CS− trials from that in the CS+ trials.

### Statistical analyses

We used the software program R (ver. 3.4.1) for all of the statistical analyses. Referring to Karos et al.^27^ for the self-report measures, we conducted a 2 × 3 [Stimulus Type (CS+ /CS−) × Block (ACQ1/ACQ2/ACQ3)] repeated measures analysis of variance (RM-ANOVA) to test for acquisition effects. Similarly, to test for extinction effects, we conducted a 2 × 3 [Stimulus Type (CS+ /CS−) × Block (ACQ3/EXT1/EXT2)] RM-ANOVA. For the duration of the acceleration and deceleration periods, we conducted a 2 × 4 [Stimulus Type (CS+ /CS−) × Block (PRC/ACQ1/ACQ2/ACQ3)] RM-ANOVA to test for acquisition effects. To test for extinction effects, we performed a 2 × 3 [Stimulus Type (CS+ /CS−) × Block (ACQ3/EXT1/EXT2)] RM-ANOVA (Table 1). Note that ACQ1 refers to the first CS+ and CS− blocks, ACQ2 refers to the second CS+ and CS− blocks, and so forth. For the time–frequency analyses of the acceleration and deceleration periods, we conducted a 3 × 4 [Frequency Band (α-band/β-band/γ-band) × Block (PRC/ACQ1/ACQ2/ACQ3)] RM-ANOVA to test for acquisition effects. To test for extinction effects, we performed a 3 × 3 [Frequency Band (α-band/β-band/γ-band) × Block (ACQ3/EXT1/EXT2)] RM-ANOVA (Table 2). We used a paired t-test for the post hoc analysis of RM-ANOVA between the acquisition phase and practice phase to test for acquisition effects, and between the extinction phase and the third block of the acquisition phase to test for extinction effects. In the RM ANOVA of the self-report measures and the duration of the acceleration and deceleration periods. we used a paired t-test for the post hoc analysis between CS+ and CS− . The Holm-Bonferroni method was used to correct for multiple testing and to keep the experiment-wise “a” value at 0.05^28^. Uncorrected degrees of freedom and corrected *p* values are given in Tables 1 and 2, as are the effect size indicated by the ƞ_p_^2^ of each ANOVA and the d-values of the post hoc t-tests.

**Table 1:** Summary data and post hoc analysis of the RM-ANOVA.

RM ANOVA	CS+				CS−				Between CS+ and CS−	
	Mean	SE	*p* value	d	Mean	SE	*p* value	d	*p* value	d
**Pain-expectancy ratings (A.U.)**										
ACQ1	6.36	0.46	–	–	2.15	0.41	–	–	< 0.01	1.99
ACQ2	7.42	0.32	< 0.05	0.57	0.95	0.25	< 0.05	− 0.75	< 0.01	4.67
ACQ3	7.48	0.38	0.07	0.57	0.82	0.27	< 0.01	− 0.82	< 0.01	4.24
EXT1	4.38	0.39	< 0.01	− 1.72	2.23	0.45	< 0.01	0.82	< 0.01	1.07
EXT2	2.57	0.42	< 0.01	− 2.64	1.48	0.34	0.07	0.46	0.10	0.56
**Fear of pain-related movement (A.U.)**										
ACQ1	4.98	0.51	–	–	2.01	0.42	–	–	< 0.01	1.32
ACQ2	5.63	0.43	0.08	0.30	1.29	0.31	0.13	− 0.42	< 0.01	2.43
ACQ3	5.97	0.44	0.16	0.45	1.00	0.25	0.08	− 0.63	< 0.01	2.92
EXT1	4.31	0.41	< 0.01	− 0.84	2.39	0.41	< 0.05	0.89	< 0.01	1.92
EXT2	2.68	0.39	< 0.01	− 1.70	1.78	0.30	0.16	0.61	0.18	0.54
**Times of acceleration phase (s)**										
PRA	0.37	0.01	–	–	0.36	0.01	–	–	0.89	0.28
ACQ1	0.39	0.02	1.00	0.19	0.37	0.01	1.00	0.05	0.69	0.21
ACQ2	0.44	0.02	< 0.05	0.92	0.34	0.01	1.00	− 0.33	< 0.01	1.61
ACQ3	0.46	0.02	< 0.01	1.07	0.34	0.01	0.48	− 0.44	< 0.01	1.97
EXT1	0.45	0.02	1.00	− 0.14	0.34	0.01	1.00	0.01	< 0.05	1.17
EXT2	0.38	0.03	< 0.05	− 0.73	0.35	0.01	0.30	0.25	0.52	0.33
**Times of deceleration phase (s)**										
PRA	0.74	0.02	–	–	0.75	0.02	–	–	1.00	0.07
ACQ1	0.84	0.03	< 0.05	0.80	0.71	0.03	0.82	− 0.36	< 0.05	1.01
ACQ2	0.86	0.04	< 0.05	0.89	0.72	0.04	0.90	− 0.24	< 0.01	0.94
ACQ3	0.85	0.04	< 0.05	0.80	0.71	0.04	0.70	− 0.31	< 0.01	0.86
EXT1	0.79	0.05	0.35	− 0.32	0.73	0.05	0.73	0.18	0.40	0.28
EXT2	0.75	0.04	< 0.05	− 0.58	0.72	0.04	0.56	0.11	0.46	0.13

The post hoc analyses of the RM-ANOVA in each CS+ and CS− were used between the second and third blocks of acquisition periods and the first block of the acquisition period in pain-expectancy rating and fear of pain-related movement, and between the acquisition periods and the practice period in times of acceleration phase and times of deceleration phase, and between the extinction periods and the third block of the acquisition period in each variable. The post hoc analyses of the RM-ANOVA also were used between CS+ and CS− . Mean: Mean value. SE: Standard error. *p* < 0.05: Statistically significant after Holm-Bonferroni correction. d: effect size of post hoc analysis. PRC: practice phase. ACQ1–3: 1st, 2nd, and 3rd blocks of the acquisition phase. EXT1–2: 1st and 2nd blocks of the extinction phase.

**Table 2:** Summary data and post hoc analysis of a one-way ANOVA.

RM ANOVA	α-band				β-band				γ-band			
	Mean	SE	*p* value	d	Mean	SE	*p* value	d	Mean	SE	*p* value	d
Wavelet coherence acceleration phase (*10^**−2**^**) (A.U.)**												
PRA	− 1.65	0.46	–	–	− 1.03	0.40	–	–	− 0.77	0.31	–	–
ACQ1	− 2.49	0.52	1.00	− 0.37	− 2.37	0.53	0.67	0.61	− 2.30	0.67	1.00	0.65
ACQ2	− 5.46	0.83	< 0.01	− 1.22	− 6.97	0.77	< 0.01	2.07	− 5.94	0.88	< 0.01	1.67
ACQ3	− 1.91	0.79	1.00	0.09	− 6.46	0.80	< 0.01	1.84	− 7.50	0.84	< 0.01	2.28
EXT1	− 0.86	0.52	1.00	0.34	− 4.85	0.73	1.00	0.42	− 5.53	1.14	0.35	0.42
EXT2	− 0.17	0.50	0.27	0.57	− 1.81	0.46	< 0.01	1.53	− 3.17	0.52	< 0.01	1.33
Deceleration phase (*10^**−2**^**) (A.U.)**												
PRA	0.02	0.33	–	–	− 0.97	0.21	–	–	0.09	0.29	–	–
ACQ1	− 4.93	0.93	< 0.01	1.60	− 5.47	0.80	< 0.01	− 1.65	− 6.55	0.81	< 0.01	− 2.33
ACQ2	− 2.20	0.46	< 0.05	1.45	− 4.13	0.64	< 0.01	− 1.42	− 4.82	0.78	< 0.01	− 1.78
ACQ3	− 2.25	0.54	< 0.01	1.25	− 2.36	0.59	1.00	− 0.68	− 3.62	0.87	< 0.05	1.22
EXT1	− 3.32	0.58	1.00	− 0.41	− 2.20	0.49	1.00	0.06	− 3.27	0.88	1.00	0.09
EXT2	− 0.23	0.38	0.06	0.92	1.84	0.55	< 0.01	1.58	0.22	0.52	< 0.01	1.14

The post hoc analysis of a one-way ANOVA was used between the acquisition periods and the practice period, and between the extinction periods and the third block of the acquisition period. Mean: Mean value. SE: Standard error. *p* < 0.05: Statistically significant after Holm-Bonferroni correction. d: effect size of post hoc analysis. PRC: practice phase. ACQ1–3: 1st, 2nd, and 3rd blocks of the acquisition phase. EXT1–2: 1st and 2nd blocks of the extinction phase.

## Results

### Pain-expectancy ratings

**Figure 3:**
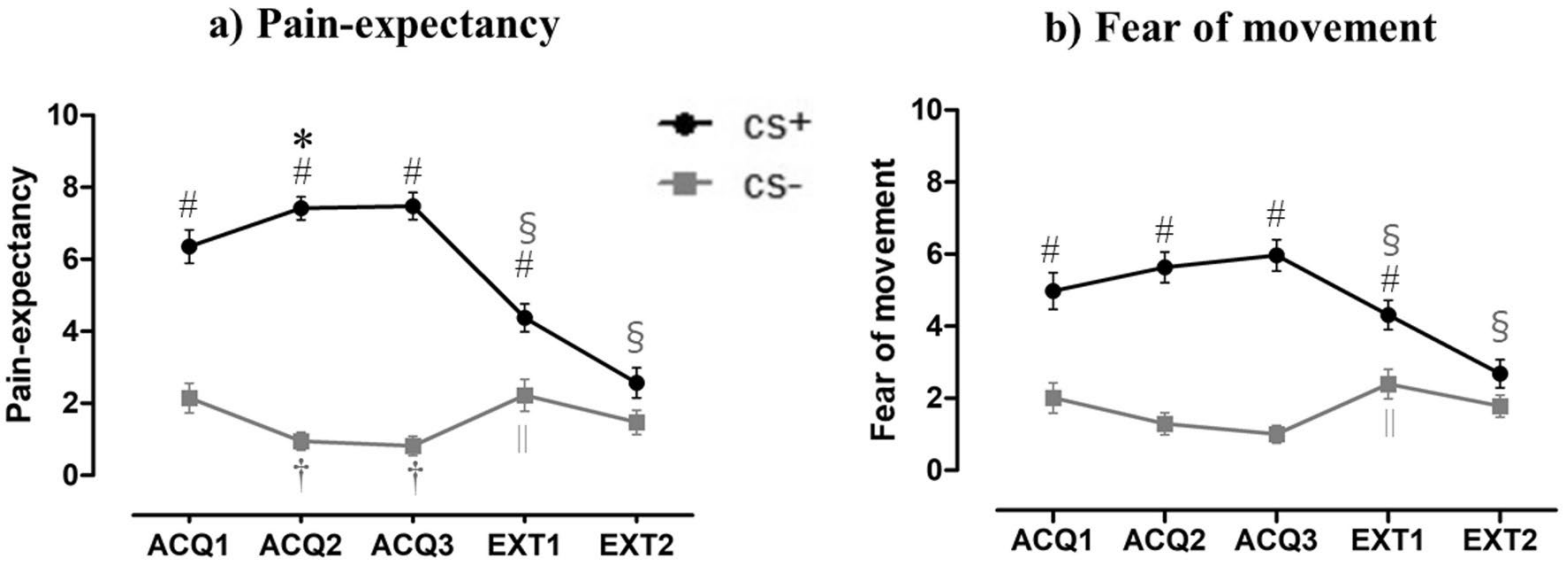
(**a**) The mean pain expectancy rating (+ SEs) for the CS movements. (**b**) The mean fear of movement rating (+ SEs) for the CS movements. ACQ1–3: 1st, 2nd, and 3rd blocks of the acquisition phase. EXT1–2: 1st and 2nd blocks of the extinction phase. #Significant difference between CS+ movement and CS− movement (*p* < 0.05). *Significant difference in CS+ movement between the practice and other phases (*p* < 0.05). †Significant difference in CS− movement between the practice and other phases (*p* < 0.05). §Significant difference in CS+ movement between the 3rd block of the acquisition phase and the other phases (*p* < 0.05). ||Significant difference in CS− movement between the 3rd block of the acquisition phase and other phases (*p* < 0.05).

During the acquisition blocks, the RM-ANOVA of the retrospective pain-expectancy ratings (Fig. 3a) revealed significant main effects for Stimulus Type (F (1, 38) = 182.80, *p* < 0.01, ƞ_p_^2^ = 0.83), but not for Block (F (2, 76) = 0.09, *p* = 0.88, ƞ_p_^2^ = 0.002). The interaction of Block × Stimulus Type was significant (F (2, 76) = 14.61, *p* < 0.01, ƞ_p_^2^ = 0.28). Regarding extinction effects, the participant's pain expectancy decreased significantly during the extinction phase, with Block (F (2, 76) = 28.32, *p* < 0.01, ƞ_p_^2^ = 0.43), but the participants were still significantly more likely to expect pain under the condition of CS+ movements compared to the condition of CS− movements at the end of the extinction phase (F (1, 38) = 57.00, *p* < 0.01, ƞ_p_^2^ = 0.60). A significant interaction of Block × Stimulus Type was revealed (F (2, 76) = 53.95, *p* < 0.01, ƞ_p_^2^ = 0.59). The results of the post hoc analyses are presented in Table 1.

### Fear of pain-related movement

During the acquisition blocks, the RM-ANOVA of the retrospective fear of movement ratings (Fig. 3b) revealed significant main effects for Stimulus Type (F (1, 38) = 64.76, *p* < 0.001, ƞ_p_^2^ = 0.63), but not for Block (F (2, 76) = 0.009, *p* = 0.97, ƞ_p_^2^ = 0.0002). The difference in the participant's fear of movement ratings between the CS+ and CS− movements changed the interaction of Block × Stimulus Type (F (2, 76) = 8.735, *p* < 0.01, ƞ_p_^2^ = 0.19). These results indicate successful fear acquisition. We next investigated extinction effects. The RM-ANOVA of the fear of movement ratings revealed significant main effects for Stimulus Type (F (1, 38) = 34.92, *p* < 0.01, ƞ_p_^2^ = 0.48) and Block (F (2, 76) = 13.01, *p* < 0.01, ƞ_p_^2^ = 0.26), plus a significant interaction of Block × Stimulus Type (F (2, 76) = 30.91, *p* < 0.01, ƞ_p_^2^ = 0.45). The results of the post hoc analyses are given in Table 1.

### The duration of the acceleration phase

**Figure 4:**
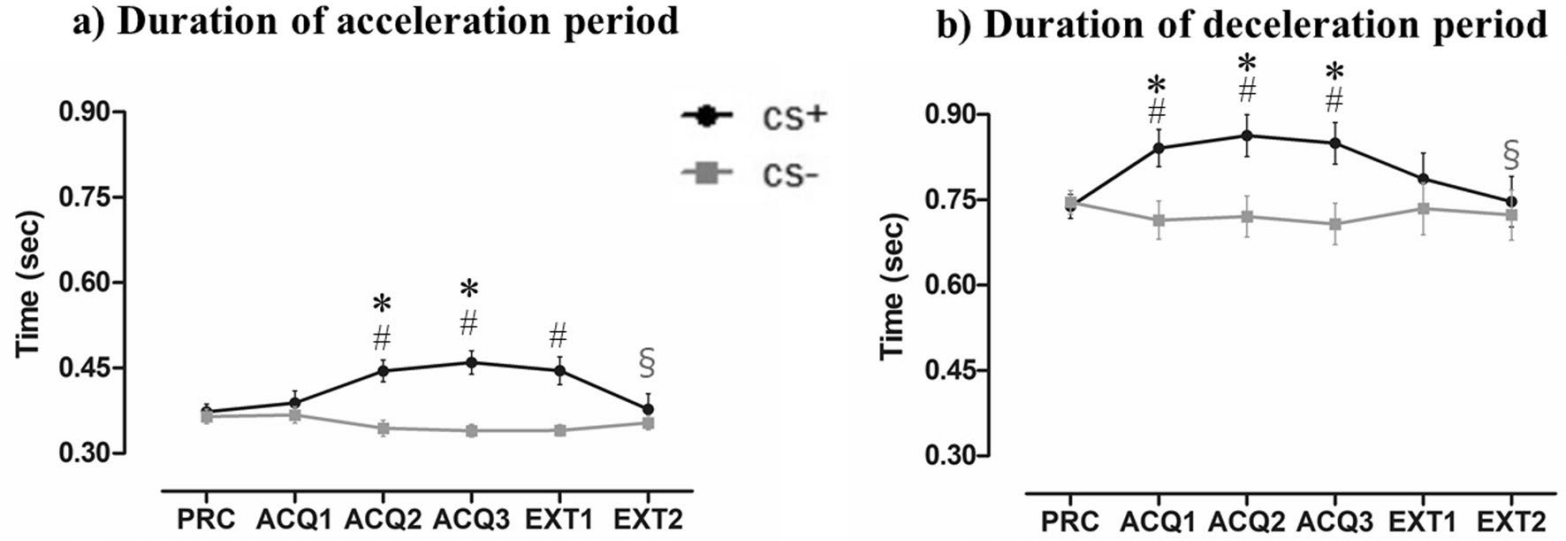
(**a**) The mean duration of the acceleration period (+ SEs) for the CS movements. (**b**) The mean duration of the deceleration period (+ SEs) for the CS movements. PRC: practice phase. ACQ1–3: 1st, 2nd, and 3rd blocks of the acquisition phase. EXT1–2: 1st and 2nd blocks of the extinction phase. #Significant difference between CS+ movement and CS− movement (*p* < 0.05). *Significant difference in CS+ movement between the practice and other phases (*p* < 0.05). §Significant difference in CS+ movement between the 3rd block of the acquisition phase and the other phases (*p* < 0.05).

During the acquisition blocks, the RM-ANOVA of the duration of the acceleration period (Fig. 4a) revealed significant main effects for Stimulus Type (F (1, 38) = 12.18, *p* < 0.01, ƞ_p_^2^ = 0.24), and Block (F (3, 114) = 5.84, *p* < 0.01, ƞ_p_^2^ = 0.13), and the interaction of Block × Stimulus Type was also significant (F (3, 114) = 17.04, *p* < 0.01, ƞ_p_^2^ = 0.31). In the extinction phase, the participants exhibited significantly longer CS+ movements compared to the CS− movements of the acceleration period, with significant main effects for Stimulus Type, F (1, 38) = 16.80, *p* < 0.01, ƞ_p_^2^ = 0.31, and Block, F (2, 76) = 7.79, *p* < 0.01, ƞ_p_^2^ = 0.17. We also observed a significant interaction of Block × Stimulus Type (F (2, 76) = 17.92, *p* < 0.01, ƞ_p_^2^ = 0.32). The results of the post hoc analyses are provided in Table 1.

### The duration of the deceleration period

During the acquisition phase, the RM-ANOVA of the duration of the acceleration period (Fig. 4b) revealed a significant main effect for Stimulus Type (F (1, 38) = 9.35, *p* < 0.01, ƞ_p_^2^ = 0.20) but not for Block (F (3, 114) = 2.22, *p* < 0.10, ƞ_p_^2^ = 0.06). A significant interaction of Block × Stimulus Type was revealed (F (3, 114) = 6.37, *p* < 0.01, ƞ_p_^2^ = 0.14). In contrast, in the extinction phase, the RM-ANOVA of the duration of the acceleration period did not reveal significant main effects for the Stimulus Type (F (1, 38) = 2.24, *p* = 0.14, ƞ_p_^2^ = 0.06) or Block (F (2, 76) = 2.37, *p* = 0.11, ƞ_p_^2^ = 0.06). The difference in the mean duration of the deceleration period between the CS+ and CS− movements changed across duration, with the interaction of Block × Stimulus Type remaining significant (F (2, 76) = 4.72, *p* < 0.05, ƞ_p_^2^ = 0.11). The post hoc analysis results are listed in Table 1.

### Time–frequency analyses

**Figure 5:**
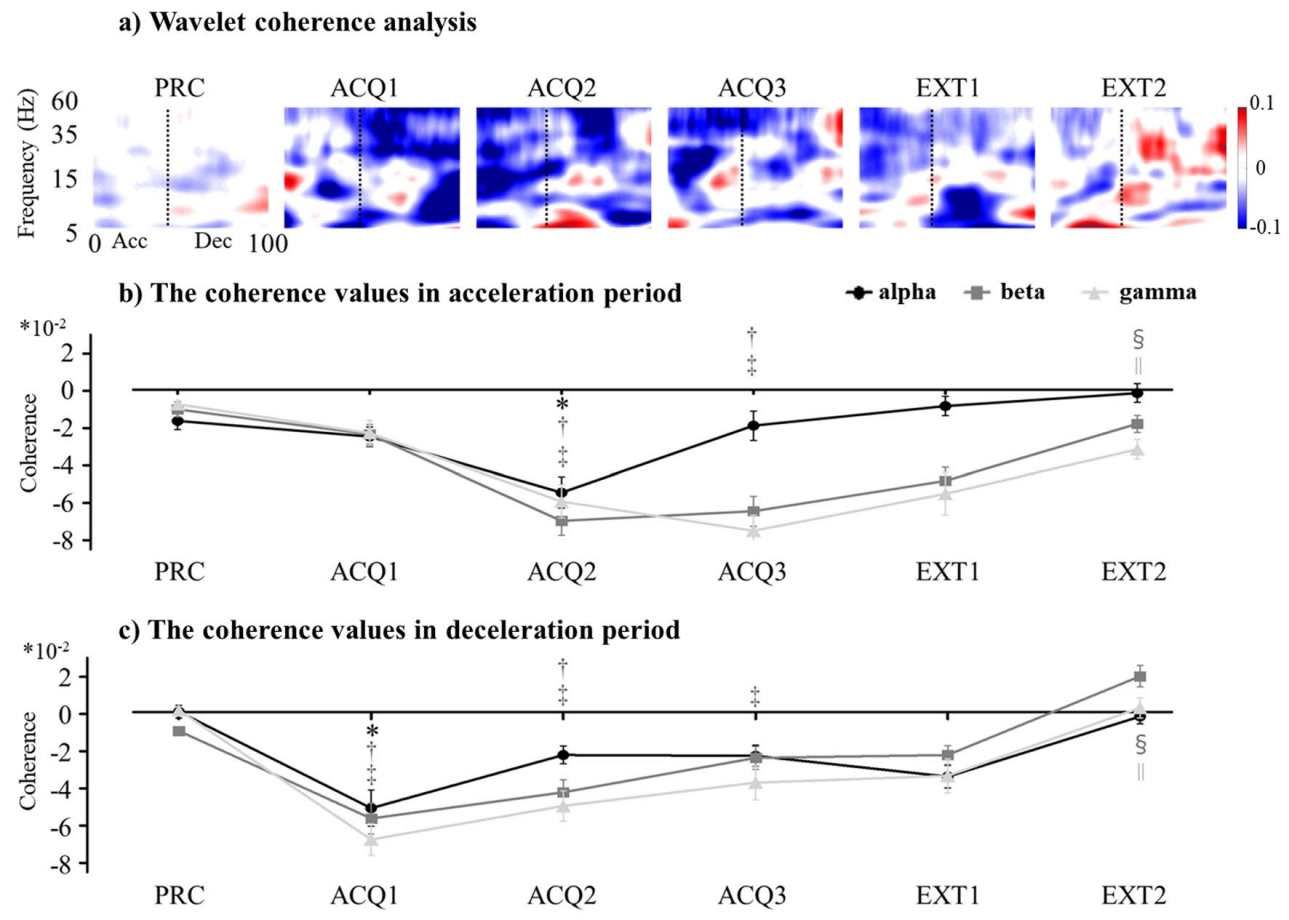
(**a**) Wavelet coherence analysis. The color bar on the right indicates the coherence value. Relative times (%) of the reaching movement are indicated on the X-axis and frequencies are illustrated on the Y-axis. Acc: Acceleration period. Dec: Deceleration period. (**b**) The coherence values in the acceleration period (ACC) (+ SEs) for the CS movements. (**c**) The coherence values in the deceleration period (DEC) (+ SEs) for the CS movements. PRC: practice phase. ACQ1–3: 1st, 2nd, and 3rd blocks of the acquisition phase. EXT1–2: 1st and 2nd blocks of the extinction phase. *Significant difference in α-bands between the practice and other phases (*p* < 0.05). †Significant difference in β-bands between the practice and other phases (*p* < 0.05). ‡Significant difference in γ-bands between the practice and other phases (*p* < 0.05). §Significant difference in β-bands between the 3rd block of the acquisition phase and the other phases (*p* < 0.05). ||Significant difference in γ-bands between the 3rd block of the acquisition phase and other phases (*p* < 0.05).

Figure 5a shows the results of the wavelet coherence analysis between the biceps and triceps brachii muscles during the reaching task. The color bar on the right indicates the coherence values. Because the time used to reach the object differed among the participants, time (X-axis) is expressed as a relative value (percentage reaching time) in Fig. 5a. The figure shows that, in general, the coherence values were lower in CS+ movements than in CS− movements.

#### Acceleration period

During the acquisition phase, the RM-ANOVA of the time–frequency analyses did not reveal a main effect for Frequency Band (F (2, 57) = 2.19, *p* = 0.12, ƞ_p_^2^ = 0.07), whereas the RM-ANOVA revealed a significant main effect for Block (F (3, 171) = 55.81, *p* < 0.01, ƞ_p_^2^ = 0.50). A significant interaction of Block × Frequency Band was revealed (F (6, 171) = 8.31, *p* < 0.01, ƞ_p_^2^ = 0.23). Regarding extinction effects, the RM-ANOVA revealed significant main effects for Frequency Band (F (2, 57) = 14.95, *p* < 0.01, ƞ_p_^2^ = 0.34) and Block (F (2, 114) = 32.05, *p* < 0.01, ƞ_p_^2^ = 0.36), but not an interaction of Block × Frequency Band (F (4, 114) = 2.34, *p* < 0.10, ƞ_p_^2^ = 0.08) (Fig. 5b). The post hoc analysis findings are shown in Table 2.

#### Deceleration period

During the acquisition phase, the RM-ANOVA of the time–frequency analyses revealed significant main effects for Frequency Band (F (2, 57) = 3.34, *p* < 0.05, ƞ_p_^2^ = 0.11) and Block (F (3, 171) = 37.63, *p* < 0.01, ƞ_p_^2^ = 0.40), but not an interaction of Block × Frequency Band (F (6, 171) = 1.38, *p* = 0.24, ƞ_p_^2^ = 0.05). Regarding extinction effects, the RM-ANOVA did not reveal a main effect for Frequency Band (F (2, 57) = 2.23, *p* = 0.12, ƞ_p_^2^ = 0.07), whereas the RM-ANOVA revealed a significant main effect for Block (F (2, 114) = 41.95, *p* < 0.01, ƞ_p_^2^ = 0.42). A significant interaction of Block × Frequency Band was not revealed (F (4, 114) = 1.29, *p* = 0.28, ƞ_p_^2^ = 0.04) (Fig. 5c). The post hoc analysis findings are shown in Table 2.

## Discussion

By undergoing our pain-related fear-conditioning paradigm, the participants learned the fear of movements in parallel with pain expectancy. Our results showed that the participants exhibited slowed goal-directed movements. We also observed that the pain-related fear conditioning resulted in increased co-contractions of agonist/antagonist muscles when the participants were making the goal-directed movements. Over the time course of the goal-directed movements, during the acceleration period the coherence values of agonist/antagonist muscles tended to decrease in the acquisition phase, whereas during the decceleration period, the coherence values tended to gradually disappear through the acquisition phase.

In general, individuals with a musculoskeletal disorder tend to make their daily movements slowly. The experience of pain after movements can slow their daily movements^29, 30^, but the daily movements remain slow even after pain alleviation^31, 32^. It has been suggested that not pain but rather the fear of movement makes daily movements slow^23, 33^. Considering these concepts, we speculate that the slowed goal-directed movements observed in the present study were related to the “acquired” fear of movement. This speculation is partially supported by the present finding of a significant negative correlation between slowed movements (= prolonged duration) and fear of pain-related movements (Suppl. Fig. S1).

In contrast, several previous studies reported that healthy participants performed movements quickly under the experimental condition of feeling fear^27, 34^. We suspect that these differences in results between previous investigations and our present study are due to the timing of the delivery of the painful stimulation. In the previous studies, in which the Pain-US was administered in the middle of the movements^27, 34^, the movements may have been fast because the safety of the subsequent movements was assured. Therefore, the ratio of the duration of the painful movement (CS+) to that of the total movement was decreased. In addition, fast movements might reflect a “get it over and done with” motor strategy when movement-related pain cannot be avoided^27, 34^. In the present study, the Pain-US was given at the completion of the participants' reaching. That is, the fear would have continued right up to the end of the reaching movement, and thus participants might have performed slowed movements such as freezing when the painful stimulation was imminent. In the defense-cascade models, an ongoing threat (i.e., performing a painful movement) would elicit an escape behavior, whereas the expectation of an imminent threat (i.e., the expectation of a painful movement) would elicit avoidance behavior^35^. In situations of unavoidable fear, fast movements are observed as escape behavior to minimize the ongoing threat event, and freezing is observed as avoidance behavior to defend the body^36, 37^. Therefore, although the changes in movements due to fear conditioning in our present study and the previous studies were both defensive responses, the movements may be faster in situations of ongoing threat, such as in the previous studies, and slower in situations of imminent threat, such as in our present paradigm.

The central processing from motor programming to motor outputs seems to be involved in the slow daily movements due to fear^38^, but there have been few investigations attempting to elucidate the mechanism by kinematic analyses. We conducted a kinematic analysis in the present study because such analysis was reported to be useful to unravel motor problems^39, 40^. In the kinematic analysis, the time course of goal-directed movements is divided into two periods: the acceleration period, which indicates the feedforward motor control, and the deceleration period, which indicates the feedback motor control. Overall, the results of our kinematic analysis revealed that the time data in the deceleration period were more promptly affected by the acquisition or the extinction of the fear-conditioning compared to those in the acceleration period. However, the time data in the acceleration period started to worsen from the second block through the acquisition phase, and these data remained worse until the end of the extinction phase. Since the feedforward motor control (reflected by the acceleration period) is involved in the process of generating movement programs, our findings may indicate that motor programs are gradually modified in relation to the acquisition of a fear of movement.

In addition, the modified motor programs were not immediately normalized but were imprinted after the acquired pain-related fear conditioning diminished. A clinical study in which patients with a musculoskeletal disorder showed ambivalently impaired goal-directed movements was able to identify abnormal cortical activities of motor programs (the supplemental motor area and premotor area)^41^. The modified feedforward motor control suggested by our present results might be explained by unnatural cortical activities of motor programs related to the participant's fear. Our analyses also revealed that coordinated contractions of agonist/antagonist muscles were more vulnerable in both the acquisition and extinction phase. The increased co-contractions of agonist/antagonist muscles are induced in response to negative emotions^42^. The increased co-contractions observed herein would then indicate “acquired” fear of movement following the pain-related fear-conditioning.

Focusing on the co-contractions, we conducted a wavelet coherent analysis in addition to the kinematic analysis. The coherence values of the α-, β-, and γ-bands in both the acceleration and deceleration periods were influenced by either the acquisition or the extinction of the pain-relevant fear conditioning. Particularly in the deceleration period, altered coherence values emerged from the first block of the acquisition phase. In contrast, in the acceleration period, the emergence of altered coherence values was delayed; these values emerged from the second block of the acquisition phase. Hence, the results of our coherent analyses of the co-contraction of agonist/antagonist muscles corresponded with the time lag observed in the kinematic analyses.

The respective frequency bands (here, the α-, β-, and γ-bands) indicate different processes of motor controls. With respect to the α-band, a reduction of the α-band intermuscular coherence of agonist/antagonist muscles is a key mechanism in the control of joint postural stabilization^26^. Excessive enhanced joint stabilization for pain-related fear might have contributed to the slow movements observed herein.

In regard to the β-band, the β-band coherence is derived specifically from cortical processing of the primary motor cortex^13, 14^. Reduced intermuscular coherence in the β-bands can lead to less-efficient motor unit recruitments (e.g., increased co-contraction of agonist–antagonist muscles and subsequent motor performance degradation)^7^. Thus, a reduction of β-band coherence would reflect maladaptive corticospinal excitability. Considering our findings of β-band coherence in conjunction with this idea, we speculate that the pain-relevant fear conditioning might impair the corticospinal excitability in both the feedforward and feedback motor controls, and the extinction of the fear might continuously impair the corticospinal excitability in the feedforward motor control but not in the feedback motor control. Since the movement-related pain expectancy can modulate the corticospinal excitability during motor preparation^34^, our findings may indicate that, compared to the feedback motor control, the feedforward motor control is more vulnerable due to the movement-related pain expectancy.

Finally, in regard to the γ-band, γ-band coherence represents inputs via subcortical pathways such as reticulospinal and/or rubrospinal tracts driven from the cerebral cortex, including the primary motor cortex^43–45^. In the present study, the γ-band coherence showed results that were similar to those of the β-band coherence, and thus γ-band coherence may be affected by the corticospinal excitability related to fear conditioning. Anecdotal evidence from a previous report suggested that the neural circuit of the central amygdala and periaqueductal gray matter coordinates panic and a rigid defensive response to the corticospinal excitability when fear is imminent^46^.

Several study limitations should be considered when interpreting the present findings. First, because the sample size was rather small, the results should be interpreted with caution and their generalizability remains unclear. Future studies with a larger sample size will be needed to confirm these findings. Second, in the acquisition phase, 3 blocks with a total of 24 CS+ trials were reinforced, whereas the usual number of trials per CS+ varies between 5 and 20^47^. In previous studies, the acquisition phase consisted of 8 trials of CS+ and 8 trials of CS− in a block to establish the fear conditioning. We set 3 blocks in order to detect any changes in the acceleration and deceleration periods of the reaching movements or changes in the wavelet coherence of the agonist/antagonist muscles between blocks in the acquisition phase. In the present study, all of the participants were told that they could stop the experiment at any time, and they were also interviewed at each acquisition phase block and asked if they wanted to continue the experiment; all of the participants completed all of the trials. However, the large number of trials per CS+ may have affected the subsequent extinction phase. We hope to reveal the influence of such stimulus numbers in a future study.

Third, we used a within-session extinction paradigm in which we checked the participants' fear of painful movements after every trial in the extinction phase. A within-session extinction reflects the process of extinction learning in which the stimulus is no longer connected with fear responses, whereas a between-session extinction reflects lasting fear alleviation based on long-term extinction learning^46^. However, the results of within-session extinction paradigm have been reported to differ from those for the between-session extinction paradigm^48, 49^. Since we did not investigate between-session extinction paradigm in this study, our results suggested short-term extinction effects, but long-term extinction learning could not be discussed. Our results should be interpreted carefully before considering their clinical applications for treating pain and fear of painful movements.

Fourth, although we revealed that slowed movements were associated with fear of pain-related movements, the participants may have been afraid to finish their movements, because the Pain-US was given at the completion of the movements. It is also possible that the movements became slow due to the participants learning that pain is not inflicted until the movement is finished (i.e., safety learning as an alternative to fear learning). To clarify this issue, further experiments with the administration of movement-related pain will be needed. Finally, we measured fear based on subjective self-reporting rather than physiological measures (e.g., skin conductance indicating an arousal condition) or behavioral indices (e.g., avoidance, withdrawal reflexes). The participants' self-reports were collected retrospectively, whereas the physiological and behavioral measures were collected during a trial. Experiments that clarify causal relationships between fear and changes in motor control by measuring physiological and behavioral measures are needed.

In summary, based on the associations of the α-, β-, γ-bands with motor control, the results obtained using our present paradigm — in which the pain-related fear was associated with goal-directed movements in healthy participants — might indicate that motor control in individuals with a musculoskeletal disorder can be explained by changes in the cortical processing of a mixture of fear, pain, and motor controls. Consequently, as results of the pain-relevant fear-conditioning, the duration of the reaching movements became longer, possibly reflecting hesitation to move. Not only were the participants' movements immediately before the painful stimuli (i.e., the deceleration period) delayed, but the movements in the acceleration period were delayed in relation to pain expectation and fear. We observed similar results regarding the co-contractions of agonist/antagonist muscular coherence, indicating that pain expectation and fear involuntarily enhance muscle co-contractions. Such negative emotional modulation might explain the protective feedforward and feedback motor abnormalities that are observed in individuals with a musculoskeletal disorder. Our results highlight the importance of evaluating the acquisition process of impaired motor control in order to identify the factors that affect the chronicity of pain.

## Supplementary Information

Supplementary Information 1.

Supplementary Information 2.
