# Supplementary figures and images for: Kinematic changes in goal-directed movements in a fear-conditioning paradigm
Source: Sci Rep. 2021 May 27;11:11162. doi: 10.1038/s41598-021-90518-7 (PMC8159940; doi:10.1038/s41598-021-90518-7)

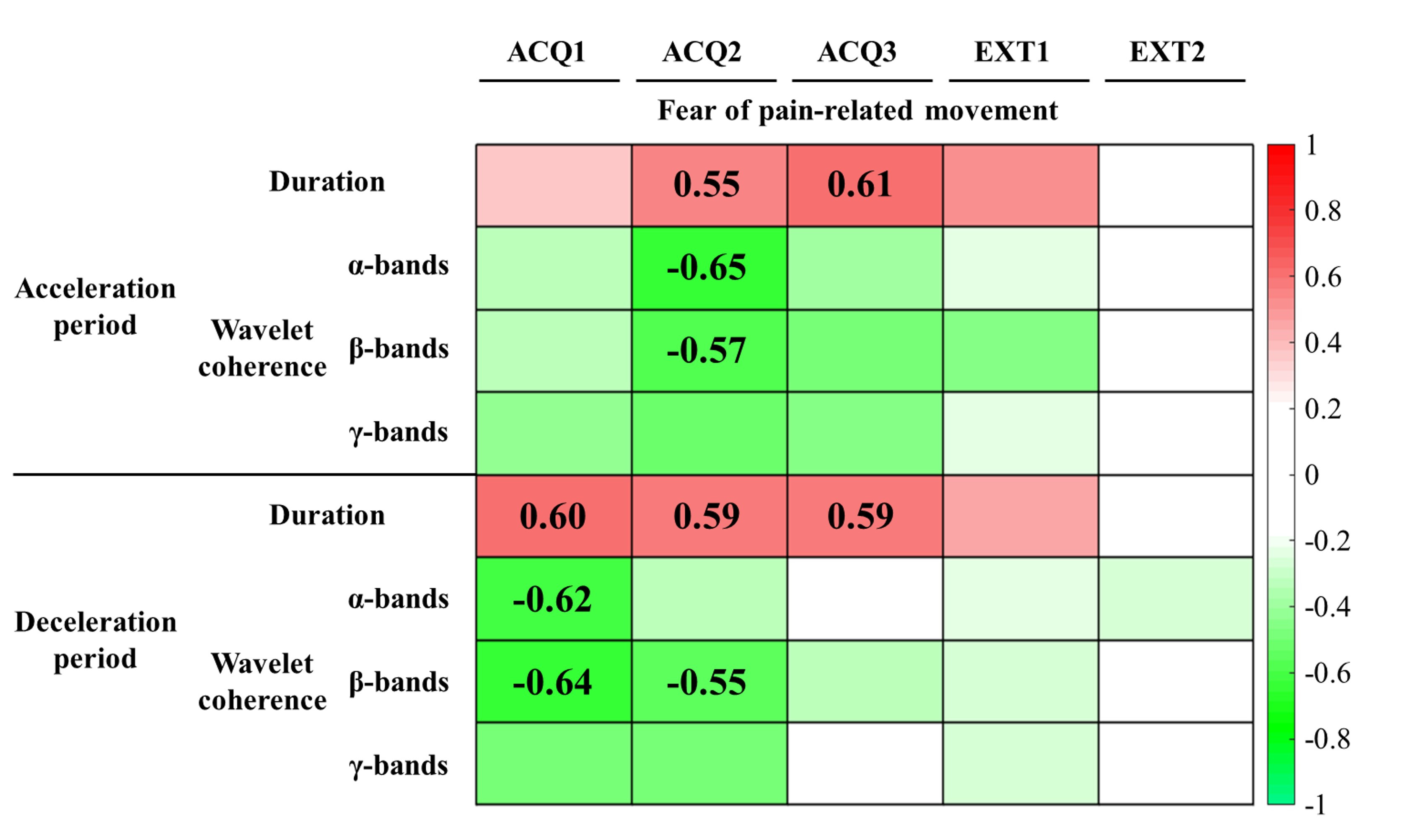

Supplement: Supplementary file 1 — Supplementary Information 1. [file 41598_2021_90518_MOESM1_ESM.jpeg]
