# Supplementary material for: Kinematic changes in goal-directed movements in a fear-conditioning paradigm
Source: Sci Rep. 2021 May 27;11:11162. doi: 10.1038/s41598-021-90518-7 (PMC8159940; doi:10.1038/s41598-021-90518-7)
Supplement: Supplementary file 2 — Supplementary Information 2. [file 41598_2021_90518_MOESM2_ESM.docx]

**Kinematic Changes in Goal-directed Movements in a Fear-conditioning Paradigm**

Yuki Nishi,^1,2,^* Michihiro Osumi,^1,3^ Masahiko Sumitani,^4^ Arito Yozu,^5^ and Shu Morioka^1,3^

^1^Graduate School of Health Sciences, Kio University, Japan

^2^Department of Rehabilitation Medicine, Nishiyamato Rehabilitation Hospital, Nara, Japan

^3^Neurorehabilitation Research Center, Kio University, Japan

^4^Department of Pain and Palliative Medicine, The University of Tokyo Hospital, Japan

^5^Center for Medical Sciences Ibaraki Prefectural University, Japan

**Supplementary Data**

Supplementary Fig. S1. Heat map showing the Pearson product-moment correlation coefficients between fear of pain-related movements and the duration of acceleration or deceleration periods and between the fear and the wavelet coherence during the acceleration or deceleration periods in acquisition and extinction phases. Each parameter using in the Pearson product-moment correlation coefficients were calculated by subtracting each parameter in the CS− trials from that in the CS + trials. Darker pixels reﬂect higher correlation values (red = positive, green = negative). The r-value is indicated only in the pixels when the correlation was signiﬁcant at p<0.05 using the Holm correction.

In the acceleration period, fear of pain-related movement had a significantly positive correlation with the duration of the acceleration period in the second and third blocks of the acquisition phase and a significantly negative correlation with the α-bands and β-bands in the wavelet coherence analysis in the second block of the acquisition phase. In the deceleration period, fear of pain-related movement had a significantly positive correlation with the duration of the deceleration period in the first, second, and third blocks of the acquisition phase and a significantly negative correlation with the α-bands in the wavelet coherence analysis in the first block of the acquisition phase, and with the β-bands in the wavelet coherence analysis in the first and second blocks of the acquisition phase.

**Supplementary Figure Legend**

**Suppl. Fig. S1.** Heat map showing the Pearson product-moment correlation coefficients between fear of pain-related movements and the duration of acceleration or deceleration periods and between the fear and the wavelet coherence during the acceleration or deceleration periods in acquisition and extinction phases. Each parameter using in the Pearson product-moment correlation coefficients was calculated by subtracting each parameter in the CS− trials from that in the CS+ trials. Darker pixels reﬂect higher correlation values (red = positive, green = negative). The r-value is indicated only in the pixels when the correlation was signiﬁcant at p<0.05 using the Holm correction.
